# Comparative sphingolipidomic analysis reveals significant differences between doxorubicin-sensitive and -resistance MCF-7 cells

**DOI:** 10.1371/journal.pone.0258363

**Published:** 2021-10-12

**Authors:** Ola D. A. Shammout, Naglaa S. Ashmawy, Sarra B. Shakartalla, Alaa M. Altaie, Mohammad H. Semreen, Hany A. Omar, Sameh S. M. Soliman

**Affiliations:** 1 College of Pharmacy, University of Sharjah, Sharjah, United Arab Emirates; 2 Research Institute for Medical and Health Sciences, University of Sharjah, Sharjah, United Arab Emirates; 3 Faculty of Pharmacy, Department of Pharmacognosy, Ain Shams University, Cairo, Egypt; 4 Pharmacy Department, City University College of Ajman, Ajman, UAE; 5 College of Medicine, University of Sharjah, Sharjah, United Arab Emirates; 6 Faculty of Pharmacy, University of Gezira, Wadmedani, Sudan; 7 Faculty of Pharmacy, Beni-Suef University, Beni-Suef, Egypt; University of Missouri Columbia, UNITED STATES

## Abstract

Drug resistance is responsible for the failure of many available anticancer drugs. Several studies have demonstrated the association between the alteration in sphingolipids (SPLs) and the development of drug resistance. To investigate the association between SPLs metabolism and doxorubicin (dox)-resistance in MCF-7 cells, a comparative sphingolipidomics analysis between dox-sensitive (parental) and -resistant MCF-7 cell lines along with validation by gene expression analysis were conducted. A total of 31 SPLs representing 5 subcategories were identified. The data obtained revealed that SPLs were clustered into two groups differentiating parental from dox-resistant cells. Eight SPLs were significantly altered in response to dox-resistance including SM (d18:1/16), SM (d18:1/24:2), SM (d18:1/24:0), SM (d18:1/20:0), SM (d18:1/23:1), HexCer (d18:1/24:0), SM (d18:1/15:0), DHSM (d18:0/20:0). The current study is the first to conclusively ascertain the potential involvement of dysregulated SPLs in dox-resistance in MCF-7 cells. SPLs metabolism in dox-resistant MCF-7 cells is oriented toward the downregulation of ceramides (Cer) and the concomitant increase in sphingomyelin (SM). Gene expression analysis has revealed that dox-resistant cells tend to escape from the Cer-related apoptosis by the activation of SM-Cer and GluCer-LacCer-ganglioside pathways. The enzymes that were correlated to the alteration in SPLs metabolism of dox-resistant MCF-7 cells and significantly altered in gene expression can represent potential targets that can represent a winning strategy for the future development of promising anticancer drugs.

## Introduction

Breast cancer is one of the most common leading causes of death in women across the world [1]. Although it represents a major health problem worldwide, survival rates continue to rise, and women are living longer. To keep this outcome, it is necessary to continue advancing our research study about the disease. The research significantly helps in the prevention, and treatment of breast cancer, and hence can improve the quality of a woman’s life. For that purpose, cell lines particularly MCF-7 are usually employed as an in vitro model to accomplish a certain level of experimental evidence. MCF-7 is the best representative for in vitro breast studies mainly because of its mammary epithelium nature that can process estrogen hormone through estrogen receptors, sensitivity to cytokeratin, and ability to form domes and monolayers [2]. It is also the first hormone-responding breast cancer cell line [2]. Although MCF-7 has been used by many research laboratories for more than 45 years, data are still generated to better understand breast cancer development and for the proper development of therapeutic strategy [3]. Thus, it constitutes a ground base for comparative research studies and data analysis not only to study the cancer pathology but also to suggest an appropriate therapy.

Drug resistance, on the other hand, is a major barrier in the efficient treatment of cancer. It is responsible for the failure of many available drugs and hence may lead to their disappearance from the market [4]. Doxorubicin (dox) is currently one of the most effective chemotherapeutic drugs used in breast cancer therapy [5]. However, a recent report has shown that approximately 50% of breast cancer patients have developed dox resistance [4]. Despite all the studies on Dox-resistance mechanisms, it is still a major unresolved problem in cancer therapy.

Recently, the role of cellular lipids in both effective therapy and resistance is drawing scientists attention [4]. Sphingolipids (SPLs) are a class of cellular lipids that play an important role in the structural integrity and fluidity of mammalian cell lipid bilayer [4]. SPLs including ceramide (Cer), sphingomyelin (SM), sphingosine-1-phophate (S1P), hexosylceramide (HexCer), sphingosine (So), and glucosylceramide (GlcCer) act as signaling molecules that contribute in the regulation of several biological processes of a cell [4]. These include cell proliferation, apoptosis, cell differentiation, cell migration, angiogenesis, autophagy and inflammation [6]. Furthermore, SPLs metabolic pathways influence cancer pathogenesis, drug resistance, and chemotherapeutics efficacy [4, 7]. The biochemical role of SPLs has been previously studied in cancer progression and development. Additionally, SPLs have been implicated in the mechanism of action of many chemotherapeutic agents [8, 9].

The core of SPLs metabolism is Cer, which is formed of a sphingosine base containing 18 carbons (d18), and an amide-linked fatty acyl chain with different number of carbons (C14-C26) [10]. Cer acts as a precursor for the synthesis of complex SPLs, such as SM, and GlcCer, which contain hydrophilic head groups [11]. Several studies have demonstrated a strong connection between the alteration in SPLs metabolism and drug resistance in human cancer cells [12, 13]. Enzymes in SPLs metabolic pathway are also involved in the regulation of many cancerous processes. Glucosylceramide synthase (GCS) is proved to be a key player in dox-resistance in various cancer types, importantly by the enzymatic conversion of Cer to GlcCer [14]. The generation of GlcCer acts as the precursor for the synthesis of other glycosphingolipids and gangliosides [15]. Low Cer levels are correlated with a higher degree of malignant progression and severity of prognosis in tumor cells [16]. Many studies proved that drug-resistant cells had 8 to 10-fold higher capacity to convert the precursor [3H]-palmitic acid to Cer and further to GluCer, than non-drug resistant counterparts [17]. This can be explained by the ability of Cer to mediate anti-proliferative pathways or inhibits pro-survival mechanisms [12]. In addition to that, Cer is shown to regulate gene expression, such as upregulates MMP-1 and hTERT [18, 19], activates COX-2 promoter [20], inhibits NF-κB activation [21], as well as induces GCS promoter by Sp1 [14]. However, the effect of Cer on multidrug resistance is still not well understood [22]. A strong association between low Cer levels and the elevation in GCS has been reported in dox-resistant cancer cells [23, 24].

Uchida et al examined the effect of dox on drug-sensitive HL-60 cells and drug-resistant HL-60/ADR (Adriamycin) cells [25]. Treatment with dox induced apoptosis and Cer production in drug-sensitive HL-60 cells, but not in drug-resistant HL-60/ADR cells. In dox-treated HL-60/ADR cells, the levels of mRNA, and protein of GCS were upregulated [25]. In a more recent study on MCF-7 cells, the effects of different doses of dox were tested [26]. They reported dose-dependent changes in SPLs levels, which include an increased level of Cer, dihydroceramide, S1P, and So, while reduced the levels of HexCer [26]. Moreover, UGCG silencing in Dox-resistant MCF-7 has restored cell sensitivity and increased endogenous ceramide and caspase-3 [27]. In contrast, UGCG-overexpressing MCF-7 cells have increased the cellular proliferation and dox-resistance accompanied by stimulation of Akt and ERK1/2 signaling pathways as well as upregulation of anti-apoptotic genes and multidrug resistance protein 1 (MDR1) [28]. Similarly, it has been shown by a sphingolpidomics analysis on ovarian cancer cells that the levels of cell membrane SPLs have been significantly altered in resistant cells when compared to sensitive cells, both treated with Taxol, although the target is β-tubulin [29]. Collectively, growing evidence suggests that alteration in SPLs metabolism is critical in the dox-resistance mechanism [27, 30, 31]. However, until now, there is no sphingolipidomics analysis study that explore the other potential pathways (other than GCS) in SPLs metabolism in dox-resistant MCF-7 cells. Therefore, a comprehensive sphingolipidomics study may help to elucidate the role of SPLs in dox-resistance in MCF-7 cells.

Based on this, we hypothesize that multiple SPLs metabolic pathways may play a role in dox resistance. Therefore, a comparative sphingolipidomics analysis between dox-resistant and parental P-MCF-7 breast cancer cells was conducted to identify the changes in SPLs metabolism that may be associated with dox-resistance mechanisms. Further, the critical genes and enzymes involved in the alteration of SPLs metabolism were investigated by qRT-PCR. The results of this study can be used for the effective development of cancer therapy.

## Materials and methods

### Chemicals

The internal standard (IS) was LIPID MAPS Mixture II dissolved in ethanol (LM-6005). IS is consisting of 25μM uncommon SPLs including Sphinganine (d17:0), Sphingosine (d17:1), Sphinganine-1-Phosphate (d17:0), Sphingosine-1-Phosphate (d17:1), Ceramide (d18:1/12:0), Ceramide-1- Phosphate (d18:1/12:0), Hexosyl Ceramide (d18:1/12:0), Lactosyl Ceramide (d18:1/12:0) and Sphingomyelin (d18:1/12:0). IS was purchased from Avanti Polar Lipids (Alabaster, AL). Doxorubicin was purchased from Sigma-Aldrich. Methanol (CH3OH), acetic acid (CH3COOH) and formic acid (HCOOH) were purchased from Fisher Scientific (Loughborough, UK). Potassium hydroxide (KOH) was purchased from HiMedia Laboratories (Mumbai, India). Isopropanol (IPA) was purchased from Merck (Darmstadt, Germany). Roswell Park Memorial Institute (RPMI) 1640 medium, Fetal Bovine Serum (FBS), penicillin-streptomycin (PS), LC-MS-grade chloroform (CHCl3) and phosphate buffered saline (PBS) were purchased from Sigma-Aldrich (St. Louis, MO, USA). MTT Assay Kit was purchased from (Abcam, United States). Primers were designed and custom ordered from Microgen Medical Equipment Est (UAE). For RNA extraction and cDNA synthesis, PureLink RNA Mini Kit (Thermo Fisher Scientific, Waltham, Massachusetts, United States) and SensiFAST™ cDNA Synthesis Kit (Bioline Reagents Ltd., London, United Kingdom) were used.

### Cell lines and culture

MCF-7 Human Breast Adenocarcinoma cell line was purchased from Cell Lines Service GmbH, (Eppelheim, Germany). The dox-resistant MCF-7 was created as previously described [32]. Parental MCF-7 cells were cultured in T-75 flasks and incubated at 37°C overnight. The cells were then treated with dox at their IC_10_. To develop resistance, the survived population was transferred to fresh clean flasks and treated gradually with increased concentrations of dox for 4 months. To maintain the resistance, dox at its IC_10_ concertation was kept in the growth media of breast cancer cells. The resistance was confirmed by MTT assay of each generation. The MCF-7 cell lines were maintained at 37°C and 5% CO_2_ in RPMI media (Sigma-Aldrich, USA) with 10% fetal bovine serum (Sigma-Aldrich, USA) and 1% penicillin/ streptomycin (Sigma-Aldrich, USA) (6).

### Methylthiazolyldiphenyl-tetrazolium bromide (MTT) viability assay

MTT assay was performed to verify the dox resistance in dox-resistant MCF-7 cell line. Dox-resistant MCF-7 cells were maintained at 37°C in a humidified incubator inclusive of 5% CO_2_ in RPMI media with 10% fetal bovine serum and 1% penicillin/ streptomycin. For the assessment of cell viability, parental and dox-resistant MCF-7 cells were seeded in 96-well plate at 5× 10^3^ cells/well and allowed to adhere for 24 h. 50 μL of MTT reagent with 50 μL serum-free media was mixed and then added to each well and incubated at 37°C for 3 h. After the removal of the solution, 150 μL DMSO was added and shaken for 15 min. The absorbance was then measured at 590 nm using a microplate reader (Varioskan Flash, Thermo Scientific). The sensitivity of parental and dox-resistant MCF-7 cell lines to dox was assayed. The IC_50_ for both cell lines were 0.5 μM and 18 μM, respectively. Dox-resistant MCF-7 is 36-fold resistant to dox when compared to parental MCF-7 cells.

### Sphingolipids (SPLs) extraction

SPLs extraction was adapted from a previously published methodology [29, 33]. First, MCF-7 cells were seeded into T-75 flasks and grown to confluence (3 million cell/mL). Cells were rinsed twice with ice-cold PBS, then scraped into a glass tube. Second, 0.5 mL MeOH, 0.25 mL CHCl_3_ and 10 μL internal standards cocktail (2.5 μM) were added consecutively. Afterward, the mixture was sonicated at room temperature for 30s and then incubated at 48°C for 12 h to extract SPLs. Third, 75 μL of KOH in MeOH (1M) was added and incubated in a shaking water bath for 2 h at 37°C to cleave any glycerolipids. After cooling and neutralization with 5% acetic acid, the solution was centrifuged, and the supernatant was dried and re-dissolved prior to LC-MS analysis [29, 33].

### LC-MS conditions

Sphingolipid analysis was performed by using a well-established LC-MS method [33], an Elute Ultra high-performance liquid chromatography coupled with quadrupole time-of-flight mass spectrometry (UHPLC-QTOF/MS) (Bruker Daltonik GmbH, Bremen, Germany) system was employed for qualitative profiling and quantitative analysis. The injection volume was 10 μL for Q-TOF. An Elute UHPLC Bruker quadrupole time-of-flight C18 column Intensity Solo C18-2 (100× 2.1mm, 1.8μm) (Bruker Daltonik GmbH, Bremen, Germany) was used to separate the endogenous SPLs. The mobile phase consisted of MeOH/H_2_O/HCOOH (60:40:0.2, v/v/v) containing 10mM NH_4_OAc. The flow rate was 0.4 mL/min, and the column temperature was maintained at 40°C for each run. A linear gradient was optimized as follows: 0–3min, 0% to 10% B; 3–5min, 10% to 40% B; 5–5.3min, 40% to 55% B; 5.3–8min, 55% to 60% B; 8–8.5min, 60% to 80% B; 8.5–10.5min, 80% to 80% B; 10.5–16min, 80% to 90% B; 16–19min, 90% to 90% B; 19–22min, 90% to 100% B, followed by washing with 100% B and equilibration with 0% B. A typical data acquisition time was 20 min. The above UHPLC system was interfaced with an ESI source. The source parameters were drying gas (N_2_), temperature 150°C, flow rate 25 μL/h, nebulizer pressure 25 psi, sheath gas (N_2_) temperature was set at 200°C with a flow rate of 25 μL/h. The scan parameters were positive ion mode over m/z 110–1300, capillary voltage 4500V, nozzle voltage 300V, fragmentor voltage 175V, skimmer voltage 65V, octopole RF peak 500V, drying gas 10L/min at 220°C. A reference solution was nebulized for continuous calibration in positive ion mode using the reference mass (m/z) 922.00979800. The acquisition and data analysis were controlled using MetaboScape 4.0 from Bruker Daltonics software (Bruker Daltonik GmbH, Bremen, Germany).

### SPLs data analysis

The data obtained from LC-MS was imported into MetaboScape 4.0 software and library (Bruker Daltonik GmbH, Bremen, Germany) for the identification of SPLs. Then, Microsoft Excel was used to collect and classify the data into tables. In order to investigate the overall differences between the parental and dox-resistant MCF-7, two multivariate analyses were carried out, namely, partial least squares–discriminant analysis (PLS-DA) and hierarchical cluster analysis (HCA). The PLS-DA was used to efficiently differentiate between the parental and dox-resistant MCF-7 cells to identify the significantly different SPLs, while HCA is an unsupervised analysis technique that classified the data into clusters. The most significantly different SPLs between the two cell lines were selected according to Variable Importance in Projection (VIP) value using MetaboAnalyst 4.0 software. VIP values higher than 1.00 were considered significant. Kyoto Encyclopedia of Genes and Genomes (KEGG) LIPIDS PATHWAY database was used to study the SPLs metabolic pathways.

### Quantitative real-time polymerase chain reaction (qRT-PCR)

Dox-sensitive (parental) and -resistant MCF-7 cell pellets were placed on ice and treated with 0.6 mL of lysis buffer and vortexed until dispersion. Total RNA was extracted using PureLink RNA Mini Kit (Thermo Fisher Scientific, Waltham, Massachusetts, United States) following the manufacturer protocol. The RNA samples were treated with DNase-I treatment (On-column PureLink® DNase, ThermoFisher, USA) solution for 15 min at room temperature to remove contaminated DNA. RNA was then eluted by adding 30 μL of RNase-free water at room temperature for 1 min followed by centrifugation at 12,000 RPM. The purity and yield of RNA were assessed by nanodrop2000 spectrophotometer (ThermoScinetific, USA). The resulting RNA was stored at -80°C until used for cDNA synthesis. cDNA was synthesized according to the protocol provided by SensiFAST™ cDNA Synthesis Kit (Bioline Reagents Ltd., London, United Kingdom). RNA (1 μg) was mixed with 4 μL 5x TransAmp Buffer, 1 μL Reverse Transcriptase and DNase/RNase-free water up to a final volume 20 μL. The samples were placed in T100™ Thermal Cycler. Cycling conditions were annealing at 25°C for15 min, reverse transcription at 42°C for 30 min, inactivation at 85°C for 15 min. QRT-PCR was performed to quantify the expression level of 14 genes encoding rate-liming enzymes known to be critical in SPLs metabolic pathways and were identified following our SPLs analysis. GADPH transcript was used as a housekeeping gene. Primers were designed and custom ordered from Microgen Medical Equipment Est (UAE). The sequences of the designed primers are listed in **Table 1**. Ensemble Genome Browser (https://asia.ensembl.org/index.html) was used for primer design. The quality parameters of the designed pPrimers were checked using Primer-BLAST available at https://www.ncbi.nlm.nih.gov/tools/primer-blast/index.cgi?ORGANISM=9606&INPUT_SEQUENCE=NM_001618.3. Oligo software (version 9.1) was used for checking the primer dimer formation. QRT- PCR was carried out on Quant Studio 3 (Thermo Fisher) using SensiFAST™ SYBR Hi-ROX kit. PCR reaction mixture was prepared by mixing 6 μL of SensiFAST^TM^ SYBR Hi-ROX, 0.48 μL forward primer (400 nM), 0.48 μL reverse primers (400 nM), 3.04 μL water, and 2 μL template. Initial steps of qRT-PCR were 2 min at 50°C for polymerase activation, followed by a 10-min hold at 95°C. Cycles (n = 40) consisted of 15 secs melt at 95°C, followed by 1-min annealing at 60°C and 20 secs extension at 72°C. The final incubation step was set at 60°C for 1 min. All samples were amplified in triplicates. Ultra-pure RNA-free water was included in the run as a negative control. The average threshold cycle (Ct) values were obtained from each reaction, and the relative expression was quantified using the 2^(−ΔΔC(T))^ method [34].

**Table 1:** List of primers used in qRT-PCR.

**Gene Symbol**	**Gene name**	**Forward primer (5′~3′)**	**Reverse primer (5′~3′)**	Amplicon length (bp)
**UGCG**	UDP-glucose ceramide glucosyltransferase	GATGTGTTGGATCAAGCAGG	TGAGTGGACATTGCAAACC	113
**CERS2**	Ceramide synthase 2	CTCTTCCTCATCGTTCGATAC	GTCAGGTAGAAATGTTCCAAGG	128
**CERS4**	Ceramide synthase 4	CATCCCTGTACTGGTGGT	CACGAAGTGGTGTATCAC	116
**SGMS1**	Sphingomyelin synthase 1	GGTCATGCTAACACTTACCTAC	GTCATGCGCTAAGAGAATACAG	124
**SGMS2**	Sphingomyelin synthase 2	CTGGAATGCATTTCCAGTG	CTGAAGAGGAAGTCTCCAC	136
**SMPD2**	Sphingomyelin phosphodiesterase\neutral sphingomyelinase 2	CTGGTGCTCCATCTAAGTGG	GGATGAACTGGGCCAATTC	130
**SMPD3**	Sphingomyelin phosphodiesterase neutral sphingomyelinase 3	CAACATGCACCCAGAAGAC	CTGACGTAGCAGTTCTTGG	135
**DEGS1**	Dihydroceramide desaturase 1	CTTCGAGTGGGTCTACAC	ACCCAACTGGGTGAGAAC	142
**DEGS2**	Dihydroceramide desaturase 2	ATGGGCCTCTCAACTGGA	AGGTGGTCGTAGTACTCG	124
**CERS5**	Ceramide synthase 5	CGAGGACAGTGGTCCTTATC	CAATCCAGCTGCTTTGACAG	117
**GBA**	Glucosylceramidase beta	CTGCTCTCAACATCCTTG	GTGCGGATGGAGAAGTCA	130
**GALC**	beta-galactosylceramidase	TCACCACTGGTCGCAAAG	GCAAAGTTTGGAGCTTCAC	118
**CERS6**	Ceramide synthase 6	CCTCTATCTCGCTTTTCC	GGAGCAATTTGTGGTCCA	123
**UGT8/CGT**	ceramide galactosyltransferase	GAGGAATCCTAACCAAACCAG	CTTCTGACAGATACTTGACACC	125
**GAPDH**	Glyceraldehyde 3-phosphate dehydrogenase	TGACTCAACACGGGAAACC	TCGCTCCACCAACTAAGAAC	100 [35, 36]

### Statistical analysis

The reports were extracted from MetaboScape 4.0 software (Bruker Daltonik GmbH, Bremen, Germany) to Microsoft Excel, where the compound concentration was calculated. SPLs identification was performed using MetaboScape 4.0 software and library (Bruker Daltonik GmbH, Bremen, Germany). The resulting table, including SPLs names, sample names, and intensity levels, was imported into MetaboAnalyst 4.0 software for multivariate statistical analysis. Partial least-squares discriminant analysis (PLS-DA) and hierarchical cluster analysis (HCA) were used to efficiently differentiate between the parental and dox-resistant MCF-7 cells. Variable Importance in Projection (VIP) value was calculated using MetaboAnalyst 4.0 software. VIP values higher than 1.00 were considered significant. A student’s *t*-test was applied to identify SPLs with statistically significant differences in intensity level between the two cell lines assuming that95% is the confidence level and 5% is false positive (false discovery rate FDR). *P*-values less than 0.05 were considered significant.

## Results

### Sphingolipids were significantly changed in dox-resistant MCF-7 compared to parental MCF-7 cells

Comparative sphingolipids (SPLs) profiling was carried out between parental and dox-resistant MCF-7 breast cancer cells using UHPLC-QTOF/MS. Compared to reference standards, 34 different SPLs were identified (**Table 2**). The identified compounds were classified into 5 subcategories, including sphingomyelins, dihydrosphingomyelins, ceramides, dihydroceramides and hexosylceramides. Herein, we have identified significant changes in SPLs contents between both cell lines, which provided an insight into a mechanism related to dox-resistance in MCF-7 cells.

**Table 2:** Identification of SPLs in parental and dox-resistant MCF-7 cells using UHPLC-Q-TOF.

Class	Name	[M+H]+m/z	RT [min]	Molecular Formula	Calculated mass	Measured Mass	Intensity (parental)	Intensity (resistant)	MS/MS Fragments (m/z)
SM	d18:1/14:0	675.5396	12.12	C37H75N2O6P	674.5363	674.53229	33728	26032.5	264.2676, 184.0732
	d18:1/16:0	703.572	13.38	C39H79N2O6P	702.5676	702.5647	337289	316080.5	264.2694, 184.0731
	d18:1/16:1	701.5569	12.32	C39H77N2O6P	700.5519	700.54958	0	45940	264.2645, 184.0732
	d18:1/17:0	717.5873	14.19	C40H81N2O6P	716.5832	716.57999	8836	23683	264.2622, 184.0731
	d18:1/18:0	731.6032	15.19	C41H83N2O6P	730.5989	730.5959333	54133.5	271639.75	264.2678, 184.0731
	d18:1/22:0	787.6647	18.16	C45H91N2O6P	786.6615	786.65746	43480	24653	264.2655, 184.0733
	d18:1/22:1	785.6512	16.83	C45H89N2O6P	784.6458	784.64391	12693	5106.5	264.2688, 184.0731
	d18:1/24:0	815.6958	20.12	C47H95N2O6P	814.6928	814.68852	0	46835.5	264.2668, 184.0730
	d18:1/24:2	811.6665	16.78	C47H91N2O6P	810.6615	810.65925	0	12368.66667	264.2702, 184.0734
	d18:1/12:0 [IS-1]	647.511	11.01	C35H71N2O6P	646.505	646.50369	31636	27597.5	264.2699, 184.0732
	d18:1/14:0	675.5396	12.12	C37H75N2O6P	674.5363	674.53229	33728	26032.5	264.2676, 184.0732
	d18:1/15:0	689.5563	12.75	C38H77N2O6P	688.5519	688.549	19384	0	264.2750, 184.0732
	d18:1/16:0	703.572	13.38	C39H79N2O6P	702.5676	702.5647	337289	316080.5	264.2694, 184.0731
	d18:1/17:0	717.5873	14.19	C40H81N2O6P	716.5832	716.57999	8836	23683	264.2622, 184.0731
	d18:1/18:0	731.6032	15.19	C41H83N2O6P	730.5989	730.5959333	54133.5	271639.75	264.2678, 184.0731
	d18:1/20:0	759.6338	16.56	C43H87N2O6P	758.6302	758.62651	33426.5	0	264.2734, 184.0731
	d18:1/22:0	787.6647	18.16	C45H91N2O6P	786.6615	786.65746	43480	24653	264.2655, 184.0733
	d18:1/23:0	801.6848	19.12	C46H93N2O6P	800.6771	800.67752	7011.3	0	264.2674, 184.0731
	d18:1/23:1	799.6669	17.23	C46H91N2O6P	798.6615	798.65961	10210.5	0	282.2457, 264.2695, 184.0731
	d18:1/24:1	813.6803	18	C47H93N2O6P	812.6771	812.67298	68022.5	57065.5	264.2697, 184.0735
DHSM	d18:0/16:0	705.5865	14.07	C39H81N2O6P	704.5832	704.57922	75576	125088.5	184.0735
	d18:0/22:0	789.6799	19.03	C45H93N2O6P	788.6771	788.67262	8472	0	184.0734
	d18:0/20:0	761.65	17.25	C43H89N2O6P	760.6458	760.64277	11162.5	0	184.0724
Cer	d18:1/16:0	538.5198	13.63	C34H67NO3	537.5121	537.51026	83232	0	264.2684
	d18:1/22:0	622.6098	17.92	C40H79NO3	621.6077	621.60398	23370	0	264.27
	d18:1/24:0	650.641	19.87	C42H83NO3	649.6373	649.63383	44372.7	66850	632.6290, 614.6156, 264.2683
	d18:1/12:0 [IS-2]	482.4574	10.99	C30H59NO3	481.4501	481.4495	28250.75	12981	264.2678
DHCer	d18:0/16:0	540.5304	14.12	C34H69NO3	539.5277	539.52313	15382	15030	266.2833
HexCer	d18:1/12:0 [IS-4]	647.5082	10.98	C36H69NO8	643.5023	643.50089	13726.8	13440.5	264.2684
	d18:1/16:0	700.571	12.73	C40H77NO8	699.5649	699.56332	44430.7	0	264.2694
	d18:1/18:0	728.6006	14.16	C42H81NO8	728.0944	727.59332	6165.3	0	
	d18:1/24:0	812.695	18.5	C48H93NO8	811.6901	811.68775	47247.5	0	632.6302, 264.2684
	d20:1/24:1	838.7109	18.38	C50H95NO8	838.2912	837.70361	18574.7	0	
C1P	d18:1/12:0 [IS-3]	562.4223	10.006	C30H60NO6P	561.4149	561.4158	13691.3	13438	264.2688
LacCer	d18:1/12:0 [IS-5]	806.5624	10.189	C42H79NO13	805.5549	805.5551	13397	13731.5	464.4472, 264.2683
Sa	d17:0 [IS-6]	288.2901	6.632	C17H37NO2	287.2829	287.2824	13711.5	14786.8	270.2794
So	d17:1 [IS-7]	286.3106	6.558	C18H39NO	285.3034	285.3032	24728.8	18328.8	268.2643
Sa1P	d17:0 [IS-8]	368.2574	6.774	C17H38NO5P	367.2504	367.2488	14679.5	28366.8	

**Fig 1:**
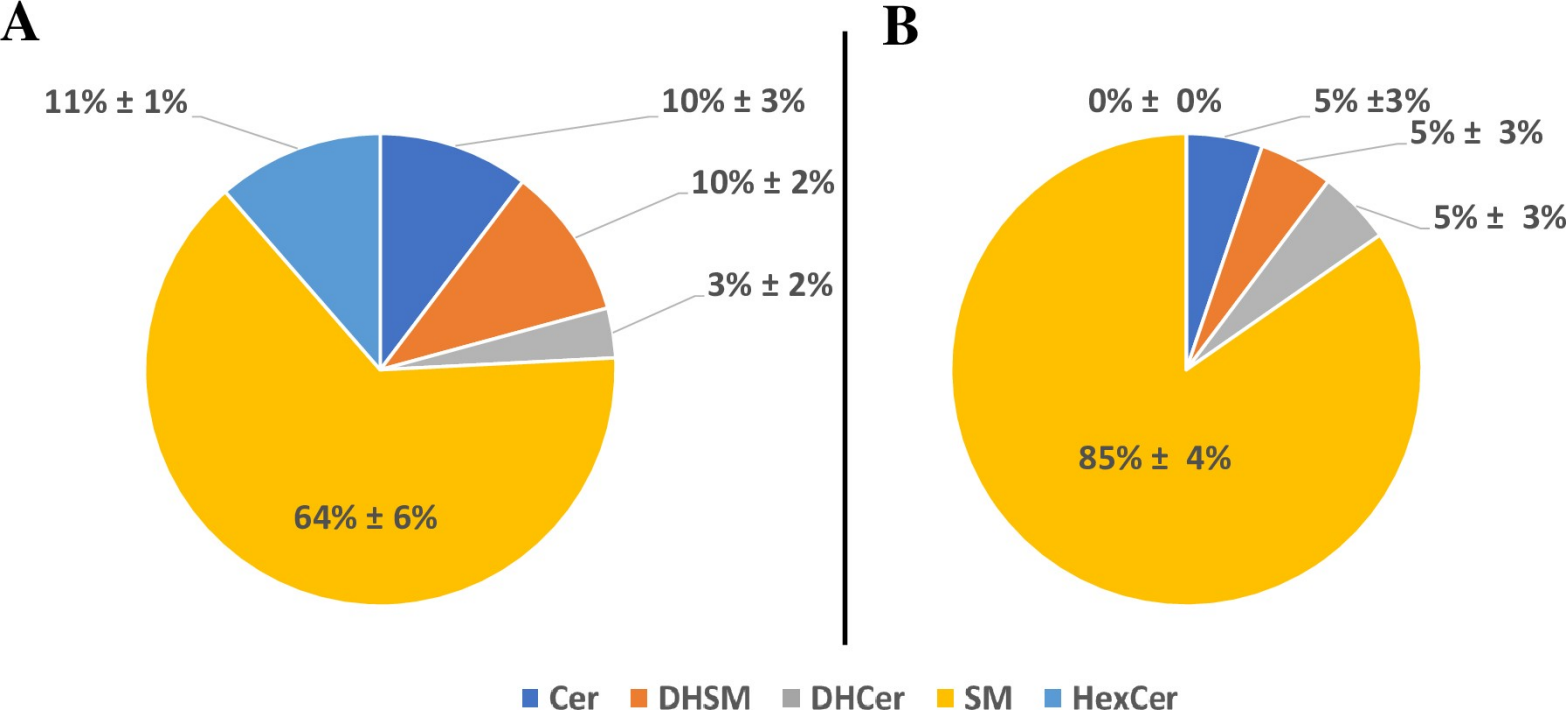
**Pie chart representing the abundance of sphingolipids in (A) Parental MCF-7 and (B) Dox-resistant MCF-7.** Identified sphingolipids included sphingomyelin (SM), dihydrosphingomyelin (DHSM), ceramide (Cer), dihydroceramide (DHCer), hexosylceramide (HexCer). The data display the mean of four replica ± Std.

**Fig 2:**
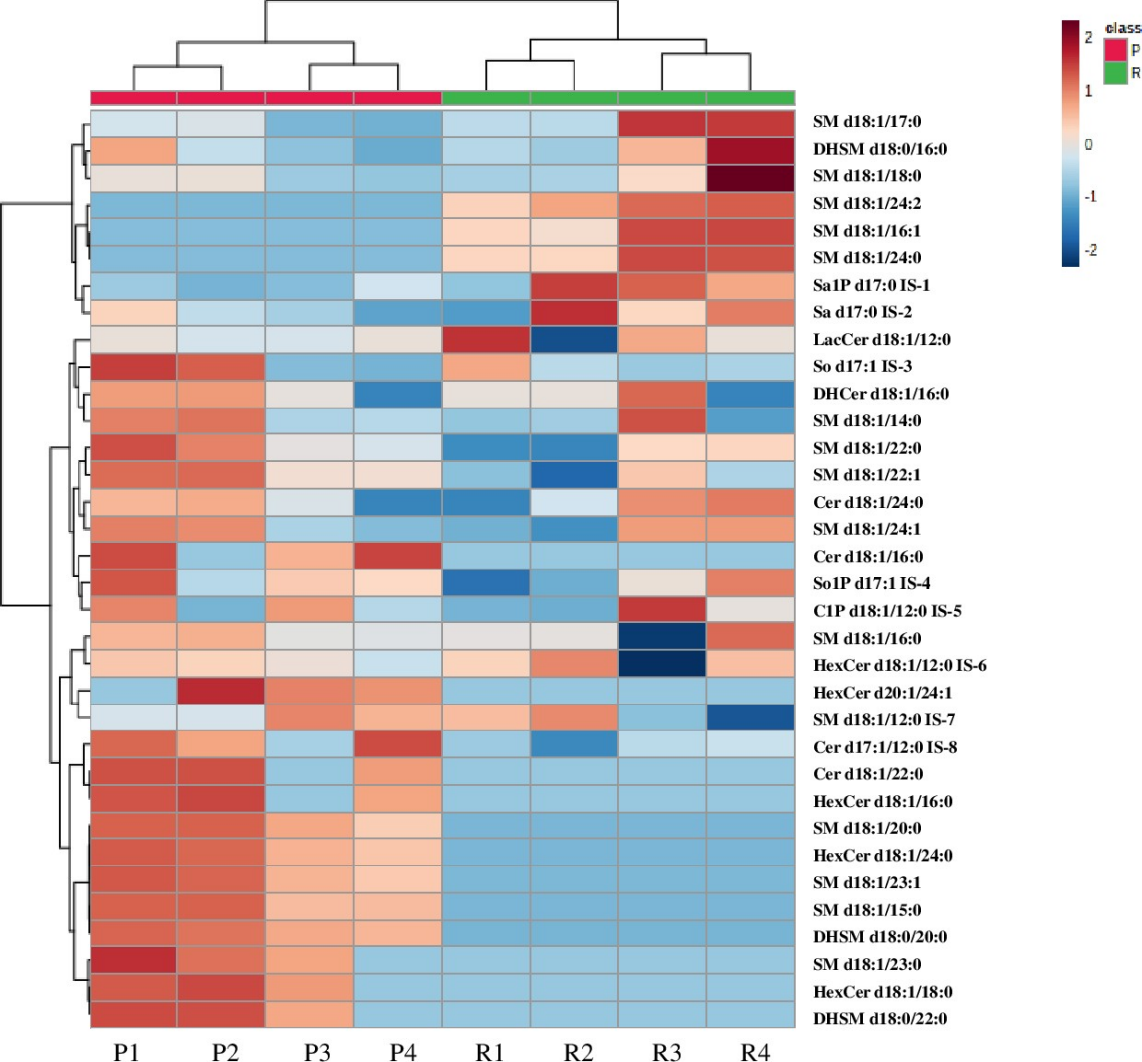
Heatmap showing the levels of SPLs in parental and dox-resistant MCF7 cells. The columns represent the type of cell line (P1-4 represents parental MCF-7 independent samples) and (R1-4 represents dox-resistant MCF-7 independent samples), while the rows represent the SPLs species. The color scale from blue to maroon represented the reduction and elevation in the levels of SPLs with respect to the median value of the intensity level. The red color indicated parental MCF-7 cells, while green indicated dox-resistant MCF-7 cells.

SM constituted the highest percentage of SPLs in both cell lines, with 64% in parental and 85% in dox-resistant MCF-7 cells (**Fig 1**). Approximately 25 different SMs were identified, including dihydrosphingomyelins (DHSMs) which contribute to 10% and 5% of all identified SPLs in the parental and dox-resistant MCF-7 cells, respectively. SMs (d18:1) was the only identified sphingoid base in both cells. The length of N-acyl chain was varied from 14 to 24, and the unsaturation degree ranged from 0 to 2. Of the 22 identified dehydrosphingomyelins, 6 SMs showed significant variable levels between both cell lines (VIP >1, *P*-value < 0.05, q<0.05) (**Fig 2** and **Table 3**). Markedly, SM (d18:1/16:1), SM (d18:1/24:0) and SM (d18:1/24:2) were only present in dox-resistant MCF-7 (VIP >1, *P*-value < 0.05, q<0.05) (**Fig 2** and **Table 3**). Further, SM d18:1/20:0, d18:1/23:1, and d18:1/23:0 and two dihydrosphingomyelins (DHSMs) including d18:1/20:0, and d18:0/22:0 were identified only in parental MCF-7. Furthermore, Cer were contributed to 10% and 5% of the total SPLs in parental MCF-7 and dox-resistant MCF-7 cells, respectively. All identified Cer were d18:1 sphingoid backbone with N-acyl chain ranged from 16 to 24 carbons. Two Cer; d18:1/16:0, and d18:1/22:0 were exclusively found in the parental cell line (**Fig 2** and **Table 3**). Notably, four different hexose-linked Cer (HexCer), including galactosylceramide and glucosylceramide, were identified only in the parental MCF-7 cells, and the most dominant was HexCer (d18:1/24:0) (VIP >1, *P*-value < 0.05, q<0.05) (**Fig 2** and **Table 3**). Out of 34 SPLs, 8 compounds were retained after FDR correction (q < 0.05) and showed significant differences between both cell lines (student’s t-test, *P*-value < 0.05) (**Table 3**).

**Table 3:** VIP scores of SPLs (VIP>1) in parental MCF-7 and dox-resistant cell lines.

SPLs names	VIP >1	Log_2_ (fold change) Resistant/Parental*	*P*-value
**SM d18:1/16:1**	1.6981	-4.5767	<0.05
**SM d18:1/24:2**	1.6322	-3.5322	<0.05
**SM d18:1/24:0**	1.6141	-3.8966	<0.05
**SM d18:1/20:0**	1.6069	3.4008	<0.05
**SM d18:1/23:1**	1.5795	3.3223	<0.05
**HexCer d18:1/24:0**	1.5643	3.2163	<0.05
**SM d18:1/15:0**	1.5135	2.9861	<0.05
**DHSM d18:0/20:0**	1.5041	2.8573	<0.05
**Cer d18:1/16:0**	1.229	3.0189	>0.05
**DHSM d18:0/22:0**	1.1949	2.7737	>0.05
**HexCer d18:1/16:0**	1.1845	2.7012	>0.05
**SM d18:1/23:0**	1.1774	2.7314	>0.05
**Cer d18:1/22:0**	1.1648	2.571	>0.05
**HexCer d18:1/18:0**	1.1586	2.5406	>0.05
**HexCer d20:1/24:1**	1.1315	2.4706	>0.05

*The log_2_ fold change Resistant/Parental shows the fold change in the intensity level when dox-resistant MCF-7 was compared to parental MCF-7 samples.

### Multivariate analysis indicated a significant alteration in unique SPLs associated with dox-resistance in MCF-7 cells

**Fig 3:**
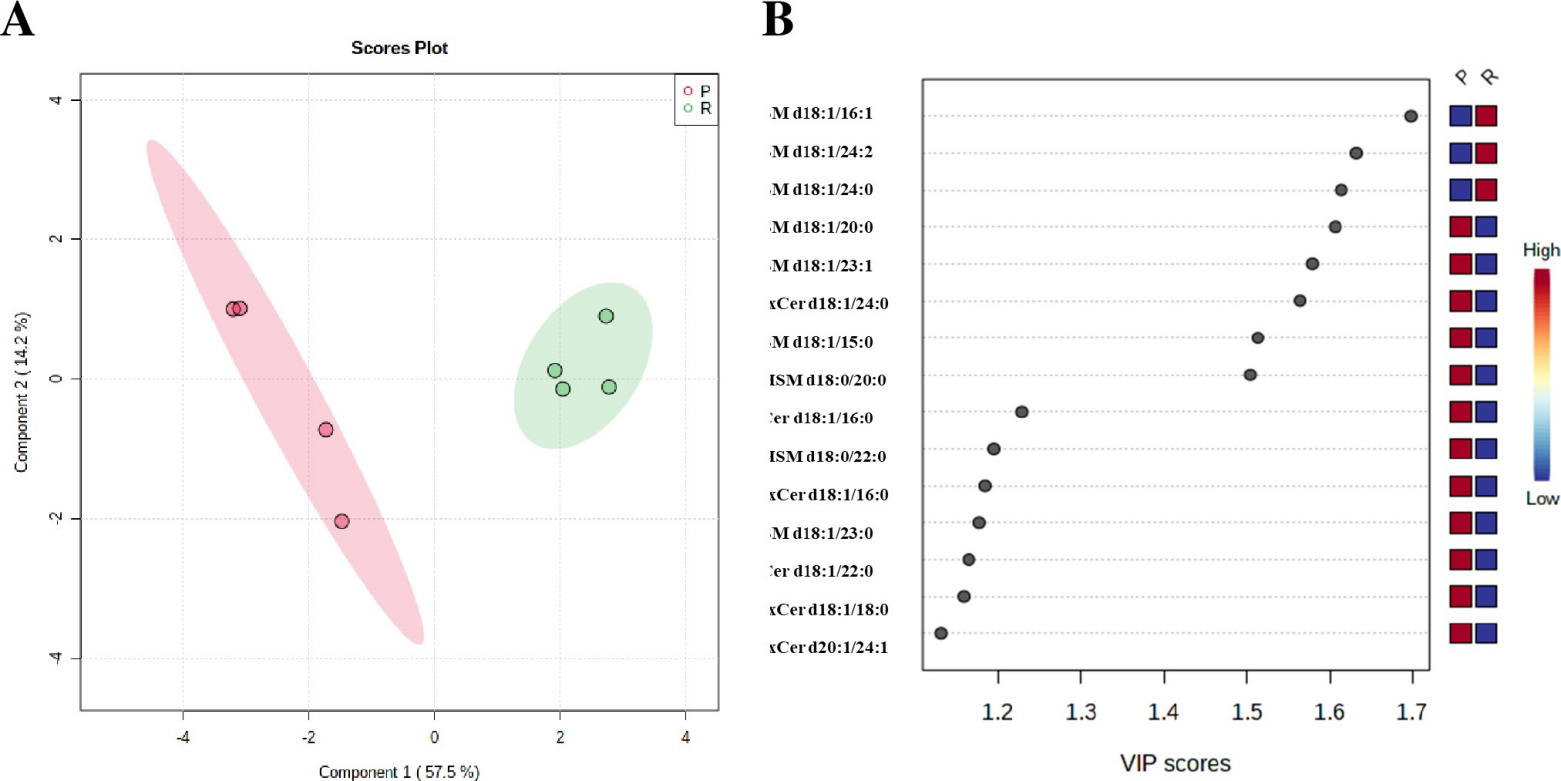
PLS-DA model for biomarker identification and selection. (**A**) 2D score plot for parental and dox-resistant MCF-7 cells. The red color indicated parental MCF-7, and green indicated dox-resistant MCF-7. (**B**) VIP score plot for parental and dox-resistant MCF-7 cells.

To investigate the overall differences between the parental and dox-resistant MCF-7 cells, multivariate analyses were carried out including HCA, and PLS-DA. HCA is an unsupervised analysis technique that was used to identify the natural patterns in the samples; thus, avoiding overfitting the sample data, while the PLS-DA was used to identify the key biomarkers that can distinguish between the two cell lines. HCA was carried out to explore the overall differences, similarities, and hidden patterns between the two cell lines. HCA classified the data into two clusters corresponding to parental and resistant cells. SPLs were clustered in the dendrogram according to their intensity levels to a hierarchical relationship that differentiated both cell types (**Fig 3**). The dendrogram showed variation in SPLs, which is most likely associated with a distinctive pattern related to each cell type and accordingly to a dox-resistance in MCF-7 cells (**Fig 2**). SM (d18:1/16:1) and SM (d18:1/24:0) showed higher abundance in dox-resistant MCF-7 compared to parental cells, indicating a significant association between alteration in SPLs and resistance mechanism in MCF-7 cells due to dox (**Fig 2**).

PLS-DA was used to extract the features that can be used to efficiently differentiate between the two cell lines. Pareto scaling and generalized log transformation function (glog) were applied to the data sets. As shown in **Fig 3A**, the variables were well-separated between the two cell lines, suggesting that this model strongly discriminates the parental from the resistant cells. Furthermore, 15 SPLs showed VIP score values greater than one; most of them were SMs (**Fig 3B**). SM d18:0/16:1, and SM d18:1/24:2 were the highest, suggesting that these SPLs could play an important role in the mechanism of dox-resistance in MCF-7 cells.

PLS-DA model is prone to the problem of overfitting, where cross-validation is an important step. The high Q2 value from the cross-validation of 0.85 coupled with the agreement between HCA and PLS-DA results is a healthy indicator of a significant statistical model. The 8 compounds with the highest VIP scores (*t*-test, *P*-value <0.05, **Table 3**) illustrated the agreement between the results obtained from the univariate analysis (student’s *t*-test) and the multivariate analysis (VIP score) (**Fig 3B**). Collectively, this significant variation in SPLs between parental and dox-resistant MCF-7 cells suggests that the alteration in SPLs metabolic pathways was most likely involved in MCF-7 resistance to dox.

### SPLs metabolism-related genes in dox-resistant MCF-7 cells were dysregulated

To validate the alterations in the levels of SPLs and to explore the importance of SPLs metabolic enzymes involved in the dox-resistance mechanism, a qRT-PCR was carried out on gene transcripts encoding 14 rate-liming enzymes in both parental and dox-resistant MCF-7 cell lines. The 14 genes were chosen based on our current results that showed differences in the levels of 15 SPLs between the two cell lines as indicated in **Tables 1 and 2** and **Figs 1–3** in association with literature search as indicated in **S1 Table**.

**Fig 4:**
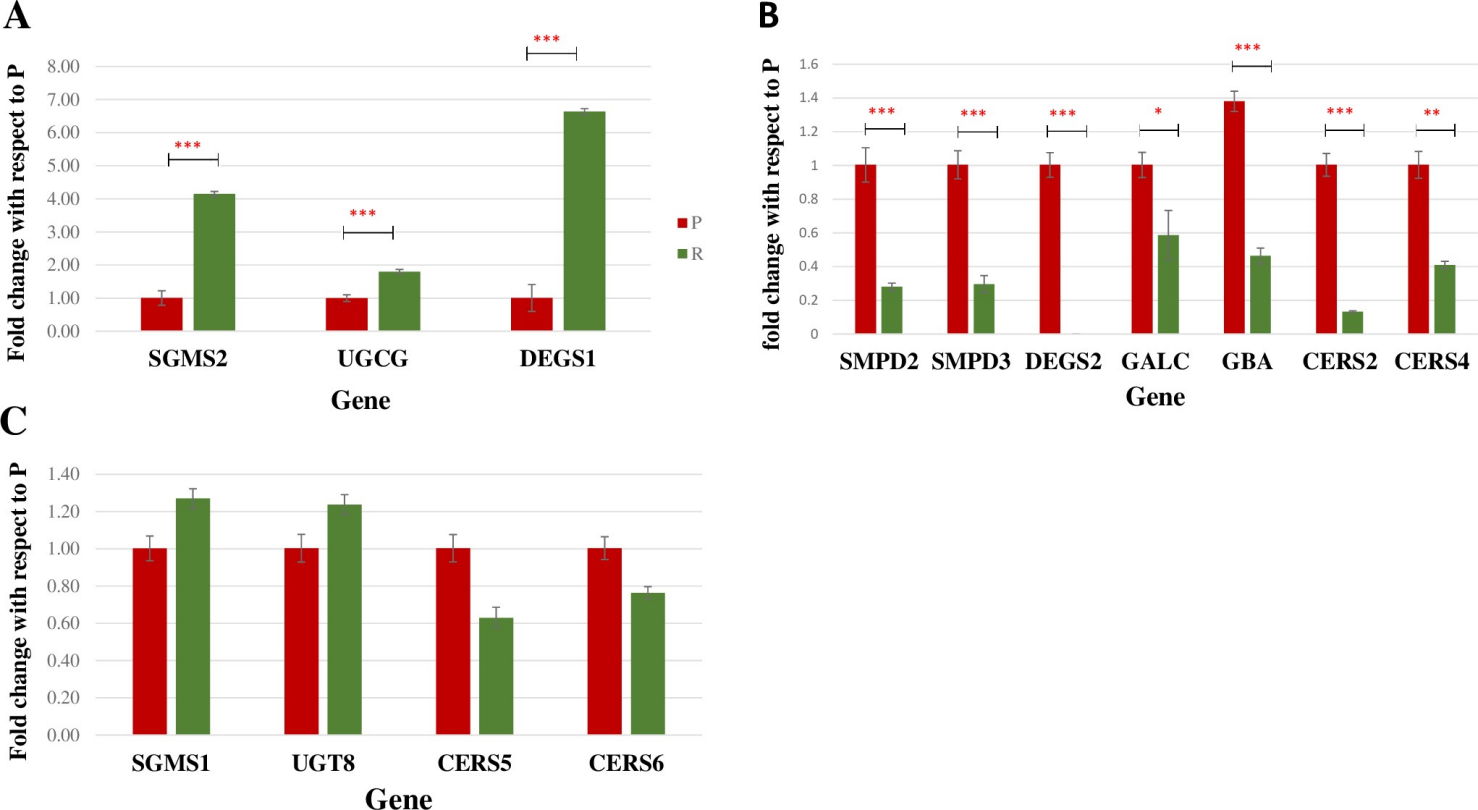
Relative mRNA expression levels of 14 genes in (P) Parental and (R) dox-resistant MCF-7 cells. (**A**) Transcripts showed a significant increase in the gene expression level in dox-resistant MCF-7. (**B**) Transcripts showed a significant increase in gene expression level in parental MCF-7. (**C**) Transcripts that showed no significant difference in gene expression between parental and dox-resistant MCF-7. *** Indicates *P*-value<0.001, ** indicates *P*-value<0.01, and * indicates *P*-value<0.05. UGCG; UDP-glucose ceramide glucosyltransferase, CERS 2; Ceramide synthase 2, CERS 4; Ceramide synthase 4, SGMS1; Sphingomyelin synthase 1, SGMS2; Sphingomyelin synthase 2, SMPD2; Sphingomyelin phosphodiesterase \neutral sphingomyelinase 2, SMPD3; Sphingomyelin phosphodiesterase neutral sphingomyelinase 3, DEGS1; Dihydroceramide desaturase 1, DEGS2; Dihydroceramide desaturase 2, CERS5; Ceramide synthase 5, GBA; Glucosylceramidase beta, GALC; beta-galactosylceramidase, CERS6; Ceramide synthase 6, UGT8/CGT; ceramide galactosyltransferase.

As demonstrated in **Fig 4**, among the 14 enzymes-coding genes, 3 genes encoding dihydroceramide desaturase 1 (DEGS1), sphingomyelin synthase 2 (SGMS2), and UDP-glucose ceramide glucosyltransferase (UGCG) were significantly upregulated in dox-resistant MCF-7 cells when compared to parental MCF-7 cells by 6.637, 4.15, and 1.8 folds, respectively (*P*-value<0.001, q<0.05) (**Fig 4A**). In contrast, a significant downregulation was exhibited in the mRNA expression level of three ceramide synthase genes (CERS 2, 4, 5) in dox-resistant MCF-7 compared to parental MCF-7 (*P*- value<0.001, q<0.05) by 0.133, 0.63, and 0.41 folds, respectively (**Fig 4B**). Further, a significant increase in the expression of neutral sphingomyelinase 2 (SMPD2) (*P*-value<0.001), neutral sphingomyelinase 3 (SMPD3) (*P*-value<0.001), dihydroceramide desaturase 2 (DEGS2) (*P*-value<0.001), and β-galactosylceramidase (GALC) (*P*-value<0.05) expression levels were observed in parental MCF-7 (**Fig 4B**), while no significant difference was observed in the mRNA expression level of glucosylceramidase beta (GBA), sphingomyelin synthase 1 (SGMS1), ceramide synthase 6 (CERS 6) and ceramide galactosyltransferase (UGT8) between the two cell lines (*P*- value>0.05, q<0.05) (**Fig 4C**).

### Proposed model of SPLs dysregulation due to dox-resistance in MCF-7 cells

**Fig 5:**
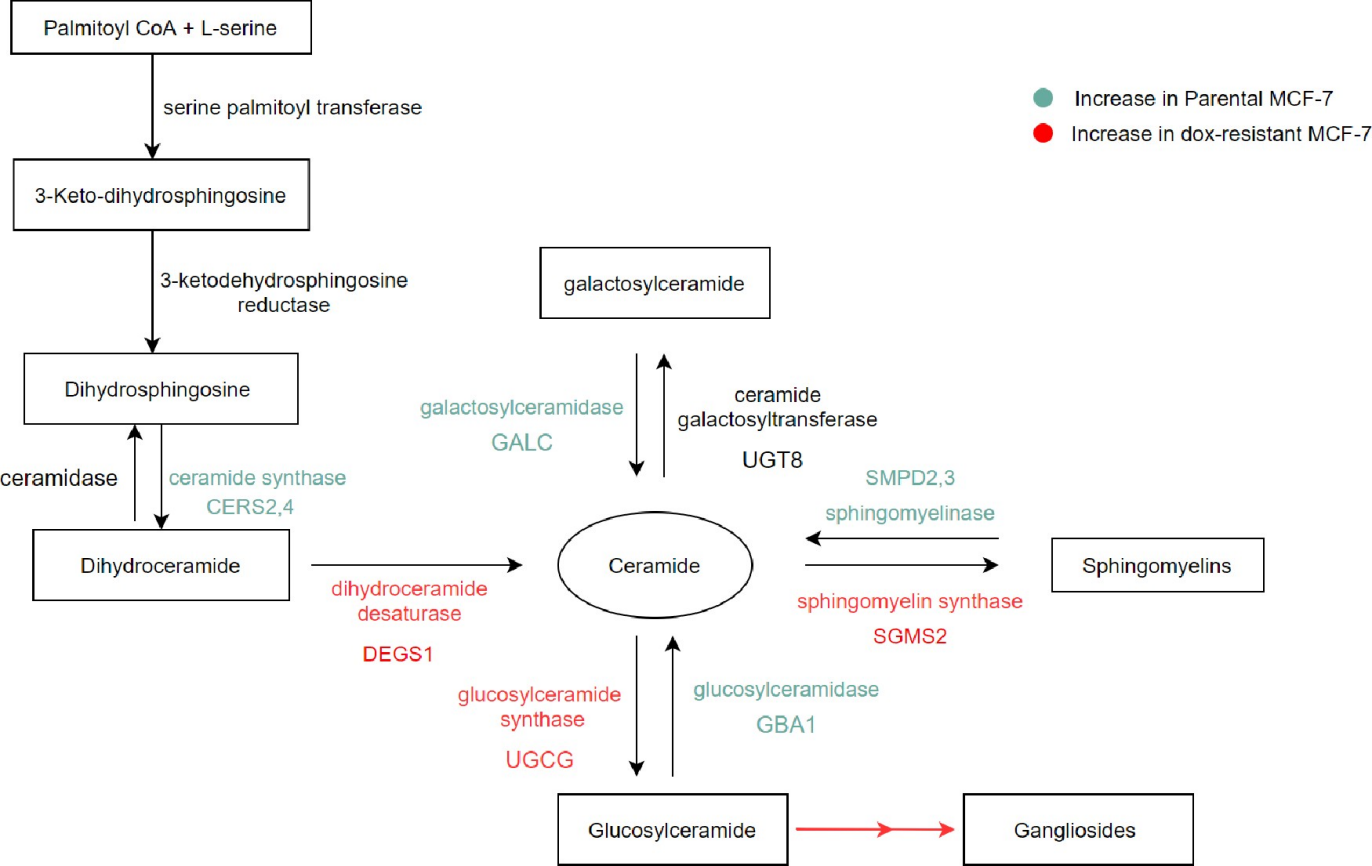
Proposed model for the alteration in SPLs metabolic pathways in parental versus dox-resistant MCF-7 cells.

Kyoto Encyclopedia of Genes and Genomes (KEGG) LIPIDS PATHWAY database in association with our current data were used to propose a model of variation in genes related to SPLs metabolism due to dox-resistance (**Fig 5**). The synthesis of Cer via de novo pathway was altered via the upregulation of dihydroceramide desaturase 1 generating Cer. The observed downregulation of Cer in dox-resistant MCF-7 cells was oriented toward two different dysregulated pathways. First, the SM-Cer pathway including the upregulation of sphingomyelin synthesis (sphingomyelin synthase 2) and the downregulation of sphingomyelinase 2/3 occurs. Second, the Cer-GluCer-ganglioside including the upregulation of glucosylceramide synthesis (glucosylceramide synthase) and the downregulation of glucosylceramidase 1 occur. This is followed by the production of ganglioside from glucosylceramide. The galactosylceramide pathway showed no significant difference, although galactosylceramidase was decreased.

## Discussion

SPLs are important building blocks that play a significant role in cell growth, proliferation, and proper functioning [12, 16]. Primarily, the role of SPLs in dox resistance in MCF-7 cells was initially investigated using comparative sphingolipidomic analysis in association with multivariate/ univariate analysis along with validation by molecular biology techniques. The data obtained revealed that SPLs were clustered into two groups differentiating parental from dox-resistant MCF-7 cells. Eight SPLs were significantly altered in response to dox resistance. These include SM d18:1/16, SM d18:1/24:2, SM d18:1/24:0, SM d18:1/20:0, SM d18:1/23:1, HexCer d18:1/24:0, SM d18:1/15:0, DHSM d18:0/20:0 with the highest VIP scores and *P*-value < 0.05, in an agreement between the univariate analysis (student’s *t*-test) and multivariate analysis (VIP score).

SM (d18:1/16:1), SM (d18:1/24:0), and SM (d18:1/24:2) were the most significantly identified SPLs in dox-resistant MCF-7, indicating their distinctive importance in the dysregulation of SPLs metabolic pathways due to dox resistance (**Fig 3** and **Table 3**). Similarly, a study comparing A2780 and Taxol-resistant A2780 (A2780T) cells has reported that SM (d18:1/16:1) and SM (d18:1/24:0) were significantly higher in A2780T [29]. Consistent with Huang et al. [29], our results showed that SM d18:1/20:0 was significantly abundant in MCF-7 compared to resistant cells. However, our data showed also that parental MCF-7 cells were significantly higher in other dihydrosphingomyelin, including SM d18:1/23:1, SM d18:1/15:0, and DHSM d18:0/20:0 (**Table 2 and Fig 3B**). Additionally, Bhadawal et al. has suggested SM d18:1/24:2 as a biomarker in breast cancer patients [35]. Generally, dihydrosphingolipids are usually found in low amounts in human cells. This can also be seen in the present study where DHSM d18:0/20:0 has exceptionally expressed in parental MCF-7 cells. Similarly, Wang et al. have suggested that the mechanism of 4-HPR–induced cytotoxicity in MDR cancer cells involves an increase in dihydrosphingolipids [36].

Importantly, this study identified rare odd carbon chains SPLs including SM d18:1/15:0, SM d18:1/17:0, SM d18:1/23:0, and SM d18:1/23:1. Notably, they are exceptionally found in low amounts in parental MCF-7 samples, thus reflecting their importance as biomarkers to indicate any changes due to altered cellular function [29]. Similarly, Huang et al. has advocated that SM d18:1/23:0, and SM d18:1/23:1 have significantly decreased in A2780T compared to A2780. Up to date, no study addresses the role of odd carbon fatty acid chains of SPLs in cancer.

Besides SM, deregulated Cer levels have shown an association with different aspects of cancer signaling and progression [37, 38]. The biological activity of Cer appears to not only rely on the fatty acid chain length, but also on the ratio of different SPLs metabolites [39]. Generally, low Cer levels have been reported as a feature of many drug-resistant cancers [40]. Here, we have identified three different Cer compounds where two of them including Cer d18:1/16:0, and Cer d18:1/22:0 were exclusively found in parental MCF-7 cells but with no statistical significance. A similar observation by Mullen et al. has confirmed the accumulation of Cer and DHCers of carbon chain length between C18-22 in MCF-7 cells [41]. Furthermore, Taxol resistant A549T cells showed lower levels of Cer 16:0 when compared to A549 cells. Markedly, upregulation of Cer 16:0 was associated with apoptosis in human colon cancer cells [42]. Other studies also showed that certain Cer compounds including Cer 16:0, accompanied with deregulation in HexCer 24:1, and SM 24:1 levels seem to affect many cellular biological processes in colon cancer, such as a cellular switch from differentiation to apoptosis [43, 44].

Dihydroceramide DHCer, on the other hand, is placed in an intermediate step in the de novo Cer synthetic pathway, catalyzed by dihydroceramide desaturase to produce Cer (**Fig 5**). Although DHCer is found in the tissues in lower concentrations, the added double bond in its structure significantly affects the membrane composition [45], fluidity, and subsequent signaling [37]. Recently, DHCer has been linked to cancer signaling and progression [46]. Many studies have confirmed the significant role of DHCer in autophagy [47]. Treatment of cancer cells with DHCer analogs or dihydrocermaide desaturase inhibitor has led to the accumulation of many endogenous DHCer compounds, mainly DHCer d18:0/16:0 and induced autophagy in cancer cells [47]. We have been able to identify the same compound in both cell lines in equal amounts. Another study has also highlighted the importance of DHCer C16:0 in glioma cells treated with DES1 inhibitor [48]. Thus, leading to a significant increase in DHCer C16:0 that resulted in ER stress and subsequent autophagy [48]. Collectively, the role of DHCer in autophagy is well-studied unlike in cancer resistance where there is sparse evidence.

Cer glycosylation is known as a crucial step in controlling Cer levels and has been highly associated with cancer resistance [49]. Four HexCer; d18:1/16:0, d18:1/18:0, d18:1/24:0, and d20:1/24:1 were identified in our study only in the parental MCF-7 cells. A study performed in human breast cancer patients has agreed with our results by showing an increase in certain compounds of HexCer (C14: 0, C16: 0, C18: 1, C18: 0, C20: 0, C22: 0, C24: 1, and C24: 0) [38]. In general, the balance between Cer and HexCers as well as the rate-limiting enzymes in Cer glycosylation such as UGCG and UGT8, are actively contributing to many aspects of cancer signaling, proliferation, and resistance [49]. Furthermore, we have reported that HexCer d18:1/24:0 was the most abundant in parental MCF-7. This can be related to a study that reported a significant decrease by 76% in HexCer d18:1/24:0 in Dox-treated MCF-7 cells and an overall dose-dependent reduction in HexCers, despite of the length of the N-acyl chain [26]. Accordingly, HexCer d18:1/24:0 seems to be targeted by Dox in MCF-7 and hence, dox-resistant cells may develop a depletion mechanism of this metabolite as a unique strategy to overcome the dox effect. Further studies are needed to confirm this hypothesis.

Collectively, dox-resistant MCF-7 cells showed significant variation in SPLs compared to parental cells indicating the importance of SPLs in cancer cell resistance mechanisms. This is considered the first report to indicate specific changes in SPLs of MCF-7 cells due to dox resistance.

To validate our findings and explore the importance of SPLs in the dox resistance mechanism, gene expression of 14 transcripts encoding rate-liming enzymes in SPLs biosynthesis, as indicated in **Fig 5** was studied. The expression of sphingomyelin synthases (SMS1, SMS2) was variable, where SMS2 was significantly upregulated in dox-resistant MCF-7 cells, while SMS1 did not show a significant difference (**Fig 4A**). SMS2 has been reported to stimulate breast cancer cell proliferation by suppressing apoptosis through a Cer-associated pathway [50]. Accordingly, SMS1 and SMS2 may hold a differential activity toward different SMs, and this can explain the significant difference in the expression of both isozymes. On the other hand, a significant downregulation was exhibited with neutral sphingomyelinase (SMPD2, and SMPD3) in dox-resistant MCF-7 cells. A study reported that the upregulation of SMPD2 was differentially induced the levels of very-long-chain (C24:1 and C24:0) Cer, which is correlated with a decrease in C24:0- and C24:1-sphingomyelins in MCF-7 cells [51]. Based on this, we suggest that SMPD 2/3 play an important role in the metabolism of SM (d18:1/24:0, and d18:1/24:2) in dox-resistant MCF-7 cells (**Fig 3** and **Table 3**). Collectively, this may explain the distinctive sphingomyelin synthesis and the consumption of Cer in dox-resistant cells, which is associated with inhibiting the pro-apoptotic effect of Cer.

The expression of three Cer synthase genes (CERS 2, 4, 5) was significantly higher in parental MCF-7 cells (**Fig 4B**). Previous studies have shown that CERS4 generates C18–C20 Cer, CERS5 and CERS6 generate C14–C16 Cer, and CERS2 selectively generates C22–C24 Cer [52]. Consistently, our results suggest that Cer d18:1/22:0, which were only found in parental cells, are mainly synthesized by the highly expressed CERS2 in parental cells. Consequently, the significant decrease of CERS in dox-resistant cells supports the dominant depletion of CERS. Gene expression of both isoforms of dihydroceramide desaturase (DeS) transcripts was analyzed because of their crucial role in controlling the balance between SPLs and dihydrosphingolipids [53]. Interestingly, our qRT-PCR results demonstrated that DeS1 was remarkably expressed in dox-resistant cells, while DeS2 was completely absent in this cell line. Consistently, resveratrol-induced autophagy in HGC-27 cells was correlated with an increase in the intracellular DHCer levels caused by the inhibition of Des1 activity [54]. Therefore, we can propose that each isoform of DeS enzyme has a distinctive action that requires further investigation to explore their roles in cancer resistance.

Several studies have demonstrated the correlation between multidrug resistance and cer glycosylation [30, 55]. UGCG converts ceramide to glucosylceramide (GluCer), which displayed elevated levels in multidrug-resistant cancer cells [56]. Similarly, we can correlate the depletion of HexCer in dox-resistant cells to the increase in UGCG gene expression, which can be explained by the activation of the ganglioside pathway [57]. This in turn enables the cancer cells to convert GluCer to gangliosides to bypass the Cer-induced apoptosis. GBA opposes the action of UGCG (**Fig 5**). siRNA for GBA1 gene was shown to induce resistance to Taxol in three different cancer cell lines [58]. In contrast, our results did not show any significant difference in the GBA gene expression in both cell lines. GALC converts GalCer to Cer, while until today, there is no report exploring the association between cancer and GALC in tumor cells. We have observed a significant downregulation of GALC expression in dox-resistant cells (**Fig 4B**), which suggests the potential correlation between GALC activity and dox resistance.

As concluding remarks, the findings from this study have conclusively ascertained the involvement of SPLs in dox resistance in MCF-7 cells. Collectively, SPLs metabolism in dox-resistant MCF-7 cells is oriented toward the downregulation of Cer and HexCer with the concomitant increase in SM. We propose that dox-resistant cells tend to escape from the Cer-related apoptosis by the activation of two different pathways: SM-Cer and GluCer-LacCer-ganglioside. The enzymes that were correlated to SPLs and significantly altered in gene expression may represent potential targets that need further investigation. Further studies on adjusting the SPLs metabolism purposively may represent a winning strategy for the future development of anticancer drugs.

## Supporting information

S1 Table Role of SPLs metabolic enzymes in cancer progression and resistance. (DOCX)

## Abbreviations

ADR: Adriamycin
C1P: Ceramide-1-phosphate
Cer: Ceramide
CERS: Ceramide synthase
COX-2: Cyclooxygenase-2
Ct: Cycle threshold
DEGS1: Dihydroceramide desaturase 1
DEGS2: Dihydroceramide desaturase 2
DHCer: Dihydroceramide
DHSM: Dihydrosphingomyelin
DMSO: Dimethyl sulfoxide
Dox: Doxorubicin
ESI: Electrospray ionization
GAPDH: Glyceraldehyde-3-Phosphate Dehydrogenase
GALC: beta-galactosylceramidase
GBA: Glucosylceramidase beta
GluCer: Glucosylceramide
HCA: Hierarchical clustering analysis
HexCer: Hexosylceramide
hTERT: Telomerase reverse transcriptase
IS: Internal standard
KEGG: Kyoto Encyclopedia of Genes and Genomes
LacCer: Lactosylceramide
MDR1: Multidrug resistance protein 1
MS/MS: Tandem mass spectrometry
MTT: Methylthiazolyldiphenyl-tetrazolium bromide
PLS-DA: Partial least squares-discriminant analysis
qRT-PCR: Quantitative Real-Time Polymerase Chain Reaction
RPM: Revolutions per minute
RPMI: Roswell Park Memorial Institute medium
Sa: Sphinganine
Sa1P: Sphinganine-1-phosphate
SGMS1: Sphingomyelin synthase 1
SGMS2: Sphingomyelin synthase 2
SM: Sphingomyelin
SMPD2: Sphingomyelin phosphodiesterase \neutral sphingomyelinase 2
SMPD3: Sphingomyelin phosphodiesterase neutral sphingomyelinase 3
So: Sphingosine
So1P: Sphingosine-1-phosphate
SPLs: Sphingolipids
UGCG/GCS: UDP-glucose ceramide glucosyltransferase/glucosylceramide synthase
UGT8/CGT: Ceramide galactosyltransferase
UHPLC-TOF/MS: Ultra High performance liquid chromatography coupled with quadrupole time-of-flight mass spectrometry
VIP: Variable importance in projection

## References

[1] Bray F, Ferlay J, Soerjomataram I, Siegel RL, Torre LA, Jemal A. Global cancer statistics 2018: GLOBOCAN estimates of incidence and mortality worldwide for 36 cancers in 185 countries. CA Cancer J Clin. 2018;68(6):394–424. Epub 2018/09/13. doi: 10.3322/caac.21492 . 30207593

[2] Camarillo IG, Xiao F, Madhivanan S, Salameh T, Nichols M, Reece LM, et al. 4—Low and high voltage electrochemotherapy for breast cancer: an in vitro model study. In: Sundararajan R, editor. Electroporation-Based Therapies for Cancer: Woodhead Publishing; 2014. p. 55–102.

[3] Comşa Ş, Cîmpean AM, Raica M. The Story of MCF-7 Breast Cancer Cell Line: 40 years of Experience in Research. Anticancer research. 2015;35(6):3147–54. Epub 2015/05/31. . 26026074

[4] Carvajal A MA, Bowen W, Wanebo H Sphingolipid biology and its role in cancer development and therapy. Clinical Case Reports and Reviews. 2016;2(1):1–4.

[5] Lao J, Madani J, Puértolas T, Alvarez M, Hernández A, Pazo-Cid R, et al. Liposomal Doxorubicin in the treatment of breast cancer patients: a review. J Drug Deliv. 2013;2013:456409. Epub 2013/05/02. doi: 10.1155/2013/456409 ; PubMed Central PMCID: PMC3619536. 23634302 PMC3619536

[6] Gomez-Larrauri A, Presa N, Dominguez-Herrera A, Ouro A, Trueba M, Gomez-Muñoz A. Role of bioactive sphingolipids in physiology and pathology. Essays in Biochemistry. 2020;64(3):579–89. doi: 10.1042/EBC20190091 32579188

[7] Ponnusamy S, Meyers-Needham M, Senkal CE, Saddoughi SA, Sentelle D, Selvam SP, et al. Sphingolipids and cancer: ceramide and sphingosine-1-phosphate in the regulation of cell death and drug resistance. Future Oncol. 2010;6(10):1603–24. Epub 2010/11/11. doi: 10.2217/fon.10.116 ; PubMed Central PMCID: PMC3071292. 21062159 PMC3071292

[8] Veldman RJ, Zerp S, van Blitterswijk WJ, Verheij M. N-hexanoyl-sphingomyelin potentiates in vitro doxorubicin cytotoxicity by enhancing its cellular influx. Br J Cancer. 2004;90(4):917–25. Epub 2004/02/19. doi: 10.1038/sj.bjc.6601581 ; PubMed Central PMCID: PMC2410169. 14970874 PMC2410169

[9] Modrak DE, Cardillo TM, Newsome GA, Goldenberg DM, Gold DV. Synergistic interaction between sphingomyelin and gemcitabine potentiates ceramide-mediated apoptosis in pancreatic cancer. Cancer Res. 2004;64(22):8405–10. Epub 2004/11/19. doi: 10.1158/0008-5472.CAN-04-2988 . 15548711

[10] Merrill AH Jr. Sphingolipid and glycosphingolipid metabolic pathways in the era of sphingolipidomics. Chem Rev. 2011;111(10):6387–422. Epub 2011/09/29. doi: 10.1021/cr2002917 ; PubMed Central PMCID: PMC3191729. 21942574 PMC3191729

[11] Ryland LK, Fox TE, Liu X, Loughran TP, Kester M. Dysregulation of sphingolipid metabolism in cancer. Cancer Biol Ther. 2011;11(2):138–49. Epub 2011/01/07. doi: 10.4161/cbt.11.2.14624 . 21209555

[12] Saddoughi SA, Song P, Ogretmen B. Roles of bioactive sphingolipids in cancer biology and therapeutics. Subcell Biochem. 2008;49:413–40. Epub 2008/08/30. doi: 10.1007/978-1-4020-8831-5_16 ; PubMed Central PMCID: PMC2636716. 18751921 PMC2636716

[13] Kreitzburg KM, van Waardenburg R, Yoon KJ. Sphingolipid metabolism and drug resistance in ovarian cancer. Cancer Drug Resist. 2018;1:181–97. Epub 2018/01/01. doi: 10.20517/cdr.2018.06 ; PubMed Central PMCID: PMC6936734. 31891125 PMC6936734

[14] Liu YY, Yu JY, Yin D, Patwardhan GA, Gupta V, Hirabayashi Y, et al. A role for ceramide in driving cancer cell resistance to doxorubicin. Faseb j. 2008;22(7):2541–51. Epub 2008/02/05. doi: 10.1096/fj.07-092981 . 18245173

[15] Futerman AH, Riezman H. The ins and outs of sphingolipid synthesis. Trends Cell Biol. 2005;15(6):312–8. Epub 2005/06/15. doi: 10.1016/j.tcb.2005.04.006 . 15953549

[16] Ogretmen B. Sphingolipid metabolism in cancer signalling and therapy. Nat Rev Cancer. 2018;18(1):33–50. Epub 2017/11/18. doi: 10.1038/nrc.2017.96 ; PubMed Central PMCID: PMC5818153. 29147025 PMC5818153

[17] Deevska GM, Dotson PP, 2nd, Karakashian AA, Isaac G, Wrona M, Kelly SB, et al. Novel Interconnections in Lipid Metabolism Revealed by Overexpression of Sphingomyelin Synthase-1. J Biol Chem. 2017;292(12):5110–22. Epub 2017/01/15. doi: 10.1074/jbc.M116.751602 ; PubMed Central PMCID: PMC5377821. 28087695 PMC5377821

[18] Reunanen N, Westermarck J, Häkkinen L, Holmström TH, Elo I, Eriksson JE, et al. Enhancement of fibroblast collagenase (matrix metalloproteinase-1) gene expression by ceramide is mediated by extracellular signal-regulated and stress-activated protein kinase pathways. J Biol Chem. 1998;273(9):5137–45. Epub 1998/03/28. doi: 10.1074/jbc.273.9.5137 . 9478967

[19] Wooten-Blanks LG, Song P, Senkal CE, Ogretmen B. Mechanisms of ceramide-mediated repression of the human telomerase reverse transcriptase promoter via deacetylation of Sp3 by histone deacetylase 1. Faseb j. 2007;21(12):3386–97. Epub 2007/06/06. doi: 10.1096/fj.07-8621com . 17548428

[20] Martin S, Phillips DC, Szekely-Szucs K, Elghazi L, Desmots F, Houghton JA. Cyclooxygenase-2 Inhibition Sensitizes Human Colon Carcinoma Cells to TRAIL-Induced Apoptosis through Clustering of DR5 and Concentrating Death-Inducing Signaling Complex Components into Ceramide-Enriched Caveolae. Cancer Research. 2005;65(24):11447–58. doi: 10.1158/0008-5472.CAN-05-1494 16357153

[21] Abboushi N, El-Hed A, El-Assaad W, Kozhaya L, El-Sabban ME, Bazarbachi A, et al. Ceramide inhibits IL-2 production by preventing protein kinase C-dependent NF-kappaB activation: possible role in protein kinase Ctheta regulation. J Immunol. 2004;173(5):3193–200. Epub 2004/08/24. doi: 10.4049/jimmunol.173.5.3193 . 15322180

[22] Yan J, Zhou Y, Chen D, Li L, Yang X, You Y, et al. Impact of mitochondrial telomerase over-expression on drug resistance of hepatocellular carcinoma. Am J Transl Res. 2015;7(1):88–99. . 25755831 PMC4346526

[23] Itoh M, Kitano T, Watanabe M, Kondo T, Yabu T, Taguchi Y, et al. Possible role of ceramide as an indicator of chemoresistance: decrease of the ceramide content via activation of glucosylceramide synthase and sphingomyelin synthase in chemoresistant leukemia. Clin Cancer Res. 2003;9(1):415–23. Epub 2003/01/23. . 12538495

[24] Liu YY, Han TY, Giuliano AE, Cabot MC. Expression of glucosylceramide synthase, converting ceramide to glucosylceramide, confers adriamycin resistance in human breast cancer cells. J Biol Chem. 1999;274(2):1140–6. Epub 1999/01/05. doi: 10.1074/jbc.274.2.1140 . 9873062

[25] Uchida Y, Itoh M, Taguchi Y, Yamaoka S, Umehara H, Ichikawa S, et al. Ceramide reduction and transcriptional up-regulation of glucosylceramide synthase through doxorubicin-activated Sp1 in drug-resistant HL-60/ADR cells. Cancer Res. 2004;64(17):6271–9. Epub 2004/09/03. doi: 10.1158/0008-5472.CAN-03-1476 . 15342415

[26] Snider JM, Trayssac M, Clarke CJ, Schwartz N, Snider AJ, Obeid LM, et al. Multiple actions of doxorubicin on the sphingolipid network revealed by flux analysis. J Lipid Res. 2019;60(4):819–31. Epub 2018/12/24. doi: 10.1194/jlr.M089714 ; PubMed Central PMCID: PMC6446699. 30573560 PMC6446699

[27] Liu YY, Han TY, Giuliano AE, Hansen N, Cabot MC. Uncoupling ceramide glycosylation by transfection of glucosylceramide synthase antisense reverses adriamycin resistance. J Biol Chem. 2000;275(10):7138–43. Epub 2000/03/04. doi: 10.1074/jbc.275.10.7138 . 10702281

[28] Wegner MS, Schömel N, Gruber L, Örtel SB, Kjellberg MA, Mattjus P, et al. UDP-glucose ceramide glucosyltransferase activates AKT, promoted proliferation, and doxorubicin resistance in breast cancer cells. Cell Mol Life Sci. 2018;75(18):3393–410. Epub 2018/03/20. doi: 10.1007/s00018-018-2799-7 . 29549423 PMC11105721

[29] Huang H, Tong TT, Yau LF, Chen CY, Mi JN, Wang JR, et al. LC-MS Based Sphingolipidomic Study on A2780 Human Ovarian Cancer Cell Line and its Taxol-resistant Strain. Sci Rep. 2016;6:34684. Epub 2016/10/06. doi: 10.1038/srep34684 ; PubMed Central PMCID: PMC5050431. 27703266 PMC5050431

[30] Gouazé V, Liu YY, Prickett CS, Yu JY, Giuliano AE, Cabot MC. Glucosylceramide synthase blockade down-regulates P-glycoprotein and resensitizes multidrug-resistant breast cancer cells to anticancer drugs. Cancer Res. 2005;65(9):3861–7. Epub 2005/05/04. doi: 10.1158/0008-5472.CAN-04-2329 . 15867385

[31] Huang W-C, Chen C-L, Lin Y-S, Lin C-F. Apoptotic Sphingolipid Ceramide in Cancer Therapy. Journal of Lipids. 2011;2011:565316. doi: 10.1155/2011/565316 21490804 PMC3066853

[32] Mdkhana B, Zaher DM, Abdin SM, Omar HA. Tangeretin boosts the anticancer activity of metformin in breast cancer cells via curbing the energy production. Phytomedicine. 2021;83:153470. Epub 2021/02/02. doi: 10.1016/j.phymed.2021.153470 . 33524703

[33] Wang JR, Zhang H, Yau LF, Mi JN, Lee S, Lee KC, et al. Improved sphingolipidomic approach based on ultra-high performance liquid chromatography and multiple mass spectrometries with application to cellular neurotoxicity. Anal Chem. 2014;86(12):5688–96. Epub 2014/05/23. doi: 10.1021/ac5009964 . 24844867

[34] Livak KJ, Schmittgen TD. Analysis of Relative Gene Expression Data Using Real-Time Quantitative PCR and the 2−ΔΔCT Method. Methods. 2001;25(4):402–8. 10.1006/meth.2001.1262 11846609

[35] Bhadwal P, Dahiya D, Shinde D, Vaiphei K, Math RGH, Randhawa V, et al. LC-HRMS based approach to identify novel sphingolipid biomarkers in breast cancer patients. Scientific Reports. 2020;10(1):4668. doi: 10.1038/s41598-020-61283-w 32170160 PMC7070000

[36] Wang H, Maurer BJ, Liu Y-Y, Wang E, Allegood JC, Kelly S, et al. N-(4-Hydroxyphenyl)retinamide increases dihydroceramide and synergizes with dimethylsphingosine to enhance cancer cell killing. Molecular Cancer Therapeutics. 2008;7(9):2967. doi: 10.1158/1535-7163.MCT-08-0549 18790777

[37] Zheng W, Kollmeyer J, Symolon H, Momin A, Munter E, Wang E, et al. Ceramides and other bioactive sphingolipid backbones in health and disease: lipidomic analysis, metabolism and roles in membrane structure, dynamics, signaling and autophagy. Biochim Biophys Acta. 2006;1758(12):1864–84. Epub 2006/10/21. doi: 10.1016/j.bbamem.2006.08.009 . 17052686

[38] Nagahashi M, Tsuchida J, Moro K, Hasegawa M, Tatsuda K, Woelfel IA, et al. High levels of sphingolipids in human breast cancer. J Surg Res. 2016;204(2):435–44. Epub 2016/08/28. doi: 10.1016/j.jss.2016.05.022 ; PubMed Central PMCID: PMC5002890. 27565080 PMC5002890

[39] Grösch S, Schiffmann S, Geisslinger G. Chain length-specific properties of ceramides. Prog Lipid Res. 2012;51(1):50–62. Epub 2011/12/03. doi: 10.1016/j.plipres.2011.11.001 22133871

[40] Senchenkov A, Litvak DA, Cabot MC. Targeting ceramide metabolism—a strategy for overcoming drug resistance. J Natl Cancer Inst. 2001;93(5):347–57. Epub 2001/03/10. doi: 10.1093/jnci/93.5.347 . 11238696

[41] Mullen TD, Jenkins RW, Clarke CJ, Bielawski J, Hannun YA, Obeid LM. Ceramide synthase-dependent ceramide generation and programmed cell death: involvement of salvage pathway in regulating postmitochondrial events. J Biol Chem. 2011;286(18):15929–42. Epub 2011/03/11. doi: 10.1074/jbc.M111.230870 ; PubMed Central PMCID: PMC3091202. 21388949 PMC3091202

[42] Machala M, Procházková J, Hofmanová J, Králiková L, Slavík J, Tylichová Z, et al. Colon Cancer and Perturbations of the Sphingolipid Metabolism. Int J Mol Sci. 2019;20(23). Epub 2019/12/06. doi: 10.3390/ijms20236051 ; PubMed Central PMCID: PMC6929044. 31801289 PMC6929044

[43] Skender B, Hofmanová J, Slavík J, Jelínková I, Machala M, Moyer MP, et al. DHA-mediated enhancement of TRAIL-induced apoptosis in colon cancer cells is associated with engagement of mitochondria and specific alterations in sphingolipid metabolism. Biochim Biophys Acta. 2014;1841(9):1308–17. Epub 2014/06/24. doi: 10.1016/j.bbalip.2014.06.005 . 24953781

[44] Tylichová Z, Slavík J, Ciganek M, Ovesná P, Krčmář P, Straková N, et al. Butyrate and docosahexaenoic acid interact in alterations of specific lipid classes in differentiating colon cancer cells. J Cell Biochem. 2018;119(6):4664–79. Epub 2017/12/24. doi: 10.1002/jcb.26641 . 29274292

[45] Lachkar F, Ferré P, Foufelle F, Papaioannou A. Dihydroceramides: their emerging physiological roles and functions in cancer and metabolic diseases. Am J Physiol Endocrinol Metab. 2021;320(1):E122–e30. Epub 2020/11/03. doi: 10.1152/ajpendo.00330.2020 . 33135459

[46] Guardiola-Serrano F, Beteta-Göbel R, Rodríguez-Lorca R, Ibarguren M, López DJ, Terés S, et al. The triacylglycerol, hydroxytriolein, inhibits triple negative mammary breast cancer cell proliferation through a mechanism dependent on dihydroceramide and Akt. Oncotarget. 2019;10(26):2486–507. Epub 2019/05/10. doi: 10.18632/oncotarget.26824 ; PubMed Central PMCID: PMC6493458. 31069012 PMC6493458

[47] Gagliostro V, Casas J, Caretti A, Abad JL, Tagliavacca L, Ghidoni R, et al. Dihydroceramide delays cell cycle G1/S transition via activation of ER stress and induction of autophagy. Int J Biochem Cell Biol. 2012;44(12):2135–43. Epub 2012/09/11. doi: 10.1016/j.biocel.2012.08.025 . 22960157

[48] Hernández-Tiedra S, Fabriàs G, Dávila D, Salanueva Í J, Casas J, Montes LR, et al. Dihydroceramide accumulation mediates cytotoxic autophagy of cancer cells via autolysosome destabilization. Autophagy. 2016;12(11):2213–29. Epub 2016/11/02. doi: 10.1080/15548627.2016.1213927 ; PubMed Central PMCID: PMC5103338. 27635674 PMC5103338

[49] Liu YY, Hill RA, Li YT. Ceramide glycosylation catalyzed by glucosylceramide synthase and cancer drug resistance. Adv Cancer Res. 2013;117:59–89. Epub 2013/01/08. doi: 10.1016/B978-0-12-394274-6.00003-0 ; PubMed Central PMCID: PMC4051614. 23290777 PMC4051614

[50] Zheng K, Chen Z, Feng H, Chen Y, Zhang C, Yu J, et al. Sphingomyelin synthase 2 promotes an aggressive breast cancer phenotype by disrupting the homoeostasis of ceramide and sphingomyelin. Cell Death & Disease. 2019;10(3):157. doi: 10.1038/s41419-019-1303-0 30770781 PMC6377618

[51] Marchesini N, Osta W, Bielawski J, Luberto C, Obeid LM, Hannun YA. Role for mammalian neutral sphingomyelinase 2 in confluence-induced growth arrest of MCF7 cells. J Biol Chem. 2004;279(24):25101–11. Epub 2004/03/31. doi: 10.1074/jbc.M313662200 . 15051724

[52] Kraveka JM, Li L, Szulc ZM, Bielawski J, Ogretmen B, Hannun YA, et al. Involvement of dihydroceramide desaturase in cell cycle progression in human neuroblastoma cells. J Biol Chem. 2007;282(23):16718–28. Epub 2007/02/07. doi: 10.1074/jbc.M700647200 ; PubMed Central PMCID: PMC2084375. 17283068 PMC2084375

[53] Siddique MM, Bikman BT, Wang L, Ying L, Reinhardt E, Shui G, et al. Ablation of dihydroceramide desaturase confers resistance to etoposide-induced apoptosis in vitro. PLoS One. 2012;7(9):e44042-e. Epub 2012/09/11. doi: 10.1371/journal.pone.0044042 . 22984457 PMC3439484

[54] Signorelli P, Munoz-Olaya JM, Gagliostro V, Casas J, Ghidoni R, Fabriàs G. Dihydroceramide intracellular increase in response to resveratrol treatment mediates autophagy in gastric cancer cells. Cancer Lett. 2009;282(2):238–43. Epub 2009/04/28. doi: 10.1016/j.canlet.2009.03.020 . 19394759

[55] Liu YY, Han TY, Giuliano AE, Cabot MC. Ceramide glycosylation potentiates cellular multidrug resistance. Faseb j. 2001;15(3):719–30. Epub 2001/03/22. doi: 10.1096/fj.00-0223com . 11259390

[56] Lavie Y, Cao H, Volner A, Lucci A, Han TY, Geffen V, et al. Agents that reverse multidrug resistance, tamoxifen, verapamil, and cyclosporin A, block glycosphingolipid metabolism by inhibiting ceramide glycosylation in human cancer cells. J Biol Chem. 1997;272(3):1682–7. Epub 1997/01/17. doi: 10.1074/jbc.272.3.1682 . 8999846

[57] Giussani P, Tringali C, Riboni L, Viani P, Venerando B. Sphingolipids: key regulators of apoptosis and pivotal players in cancer drug resistance. Int J Mol Sci. 2014;15(3):4356–92. Epub 2014/03/15. doi: 10.3390/ijms15034356 ; PubMed Central PMCID: PMC3975402. 24625663 PMC3975402

[58] Swanton C, Marani M, Pardo O, Warne PH, Kelly G, Sahai E, et al. Regulators of Mitotic Arrest and Ceramide Metabolism Are Determinants of Sensitivity to Paclitaxel and Other Chemotherapeutic Drugs. Cancer Cell. 2007;11(6):498–512. 10.1016/j.ccr.2007.04.011 17560332

