# Supplementary material for: Comparative sphingolipidomic analysis reveals significant differences between doxorubicin-sensitive and -resistance MCF-7 cells
Source: PLoS One. 2021 Oct 12;16(10):e0258363. doi: 10.1371/journal.pone.0258363 (PMC8509934; doi:10.1371/journal.pone.0258363)
Supplement: S1 Table — (DOCX) [file pone.0258363.s001.docx]

**S1 Table. Role of SPLs metabolic enzymes in cancer progression and resistance.**

| **Enzyme name** | **Gene*** | **Role in cancer/ rationale of gene selection** | **References** |
| --- | --- | --- | --- |
| Sphingomyelin synthase1 | SGMS1 | - Metastatic melanoma: SGMS1 downregulation leads to worse prognosis. - Leukemia: SGMS1 upregulation increases cell proliferation | [1]  [2] |
| Sphingomyelin synthase 2 | SGMS2 | Breast cancer: Increase roliferation and migration. Reduce apoptosis. | [3] |
| Neutral sphingomyelinase 2 | SMPD2 | - Breast cancer: SMPD2 upregulation causes increase in very long chain ceramides (C24:1 and C24:0), reduction in sphingomyelins (C24:0 and C24:1) - SMPD2 downregulation causes increase in apoptosis. | [4]  [5] |
| Ceramide synthases | CerS2 CerS4  CerS5  CerS6 | - Multiple cancers types:Dysregulation of CerS activity has been associated with: - cell invasion - proliferation - apoptosis - epithelial-mesenchymal transition - CerS4 generates C18–C20 ceramides - CerS5 and CerS6 generate C14–C16 ceramides - CerS2 generates C22–C24 ceramides | [6]  [7]  [8]  [9] |
| Dihydroceramide desaturase 1 | DEGS1 | Ablation of DEGS1 Confers Resistance to Etoposide-Induced Apoptosis in Vitro. | [10] |
| Dihydroceramide desaturase 2 | DEGS2 | Gastric cancer: upregulation of DEGS2 induced autophagy. | [11] |
| Glucosylceramide synthase | GCS or UGCG | - Dox-resistant MCF-7: Silencing of UGCG can restore cell sensitivity and increase the endogenous ceramide and caspase-3. - Overexpression of UGCG increased the cellular proliferation and doxorubicin resistance and inhibited apoptosis. | [12]  [13] |
| Glucosylceramidase | GBA | Breast cancer: Silencing of GBA gene induced resistance to paclitaxel. | [14] |
| UDP-galactose:ceramide galactosyltransferase | UGT8 | Breast cancer: upregulation of UGT8 in breast cancer inhibited apoptosis. | [15, 16] |
| Β-galactosylceramidase | GALC | Colorectal Cancer: overexpression of GALC significantly increased the proliferation. | [17] |

*The aforementioned 14 genes are encoding rate-liming enzymes in SPLs biosynthesis, which have been selected for the gene expression analysis.

**References**

1. Bilal F, Montfort A, Gilhodes J, Garcia V, Riond J, Carpentier S, et al. Sphingomyelin Synthase 1 (SMS1) Downregulation Is Associated With Sphingolipid Reprogramming and a Worse Prognosis in Melanoma. Frontiers in Pharmacology. 2019;10(443). doi: 10.3389/fphar.2019.00443.

2. Moorthi S, Burns TA, Yu GQ, Luberto C. Bcr-Abl regulation of sphingomyelin synthase 1 reveals a novel oncogenic-driven mechanism of protein up-regulation. Faseb j. 2018;32(8):4270-83. Epub 2018/03/14. doi: 10.1096/fj.201701016R. PubMed PMID: 29533737; PubMed Central PMCID: PMCPMC6044059.

3. Zheng K, Chen Z, Feng H, Chen Y, Zhang C, Yu J, et al. Sphingomyelin synthase 2 promotes an aggressive breast cancer phenotype by disrupting the homoeostasis of ceramide and sphingomyelin. Cell Death & Disease. 2019;10(3):157. doi: 10.1038/s41419-019-1303-0.

4. Marchesini N, Osta W, Bielawski J, Luberto C, Obeid LM, Hannun YA. Role for mammalian neutral sphingomyelinase 2 in confluence-induced growth arrest of MCF7 cells. J Biol Chem. 2004;279(24):25101-11. Epub 2004/03/31. doi: 10.1074/jbc.M313662200. PubMed PMID: 15051724.

5. Fan SH, Wang YY, Lu J, Zheng YL, Wu DM, Zhang ZF, et al. CERS2 suppresses tumor cell invasion and is associated with decreased V-ATPase and MMP-2/MMP-9 activities in breast cancer. J Cell Biochem. 2015;116(4):502-13. Epub 2014/09/13. doi: 10.1002/jcb.24978. PubMed PMID: 25213553.

6. Chen J, Li X, Ma D, Liu T, Tian P, Wu C. Ceramide synthase-4 orchestrates the cell proliferation and tumor growth of liver cancer in vitro and in vivo through the nuclear factor-κB signaling pathway. Oncol Lett. 2017;14(2):1477-83. Epub 2017/08/10. doi: 10.3892/ol.2017.6365. PubMed PMID: 28789368; PubMed Central PMCID: PMCPMC5529835.

7. Suzuki M, Cao K, Kato S, Komizu Y, Mizutani N, Tanaka K, et al. Targeting ceramide synthase 6-dependent metastasis-prone phenotype in lung cancer cells. J Clin Invest. 2016;126(1):254-65. Epub 2015/12/10. doi: 10.1172/jci79775. PubMed PMID: 26650179; PubMed Central PMCID: PMCPMC4701566.

8. Edmond V, Dufour F, Poiroux G, Shoji K, Malleter M, Fouqué A, et al. Downregulation of ceramide synthase-6 during epithelial-to-mesenchymal transition reduces plasma membrane fluidity and cancer cell motility. Oncogene. 2015;34(8):996-1005. Epub 2014/03/19. doi: 10.1038/onc.2014.55. PubMed PMID: 24632610.

9. Kraveka JM, Li L, Szulc ZM, Bielawski J, Ogretmen B, Hannun YA, et al. Involvement of dihydroceramide desaturase in cell cycle progression in human neuroblastoma cells. J Biol Chem. 2007;282(23):16718-28. Epub 2007/02/07. doi: 10.1074/jbc.M700647200. PubMed PMID: 17283068; PubMed Central PMCID: PMCPMC2084375.

10. Siddique MM, Bikman BT, Wang L, Ying L, Reinhardt E, Shui G, et al. Ablation of dihydroceramide desaturase confers resistance to etoposide-induced apoptosis in vitro. PLoS One. 2012;7(9):e44042-e. Epub 2012/09/11. doi: 10.1371/journal.pone.0044042. PubMed PMID: 22984457.

11. Signorelli P, Munoz-Olaya JM, Gagliostro V, Casas J, Ghidoni R, Fabriàs G. Dihydroceramide intracellular increase in response to resveratrol treatment mediates autophagy in gastric cancer cells. Cancer Lett. 2009;282(2):238-43. Epub 2009/04/28. doi: 10.1016/j.canlet.2009.03.020. PubMed PMID: 19394759.

12. Liu YY, Han TY, Giuliano AE, Hansen N, Cabot MC. Uncoupling ceramide glycosylation by transfection of glucosylceramide synthase antisense reverses adriamycin resistance. J Biol Chem. 2000;275(10):7138-43. Epub 2000/03/04. doi: 10.1074/jbc.275.10.7138. PubMed PMID: 10702281.

13. Wegner MS, Schömel N, Gruber L, Örtel SB, Kjellberg MA, Mattjus P, et al. UDP-glucose ceramide glucosyltransferase activates AKT, promoted proliferation, and doxorubicin resistance in breast cancer cells. Cell Mol Life Sci. 2018;75(18):3393-410. Epub 2018/03/20. doi: 10.1007/s00018-018-2799-7. PubMed PMID: 29549423.

14. Swanton C, Marani M, Pardo O, Warne PH, Kelly G, Sahai E, et al. Regulators of Mitotic Arrest and Ceramide Metabolism Are Determinants of Sensitivity to Paclitaxel and Other Chemotherapeutic Drugs. Cancer Cell. 2007;11(6):498-512. doi: <https://doi.org/10.1016/j.ccr.2007.04.011>.

15. Owczarek TB, Suchanski J, Pula B, Kmiecik AM, Chadalski M, Jethon A, et al. Galactosylceramide affects tumorigenic and metastatic properties of breast cancer cells as an anti-apoptotic molecule. PLoS One. 2013;8(12):e84191-e. doi: 10.1371/journal.pone.0084191. PubMed PMID: 24391908.

16. Yang F, Foekens JA, Yu J, Sieuwerts AM, Timmermans M, Klijn JGM, et al. Laser microdissection and microarray analysis of breast tumors reveal ER-α related genes and pathways. Oncogene. 2006;25(9):1413-9. doi: 10.1038/sj.onc.1209165.

17. Yang M, Jiang Z, Yao G, Wang Z, Sun J, Qin H, et al. GALC Triggers Tumorigenicity of Colorectal Cancer via Senescent Fibroblasts. Frontiers in Oncology. 2020;10(380). doi: 10.3389/fonc.2020.00380.
